# Effect of COVID-19 on liver abnormalities: a systematic review and meta‐analysis

**DOI:** 10.1038/s41598-021-89513-9

**Published:** 2021-05-19

**Authors:** Khalid Bzeizi, Maheeba Abdulla, Nafeesa Mohammed, Jehad Alqamish, Negar Jamshidi, Dieter Broering

**Affiliations:** 1 Department of Liver & Small Bowel Transplantation & Hepatobiliary-Pancreatic Surgery, King Faisal Specialist Hospital & Research Center, P.O. Box 3354, Riyadh, 11211 Saudi Arabia; 2 Internal Medicine Department, Salmaniya Medical Complex, Manama, Bahrain; 3 Ibn AlNafees Hospital, Manama, Bahrain; 4 School of Science, RMIT University, Melbourne, VIC 3000 Australia; 5 Organ Transplant Center & Department of Surgery, King Faisal Specialist Hospital & Research Centre (Gen. Org) MBC 96, AlFaisal University, P.O. Box 3354, Riyadh, 11211 Saudi Arabia

**Keywords:** Gastrointestinal diseases, Liver diseases

## Abstract

Emerging evidence suggest association of severe acute respiratory syndrome coronavirus 2 (SARS-CoV-2) infection with the development of many liver abnormalities. The overarching aim of this study was therefore to assess the available evidence on the clinical effects of SARS-CoV-2 on the profiles of liver chemistries and coagulation in COVID-19 diagnosed patients. We considered all study designs including epidemiological and observational that reported liver function test abnormalities in patients confirmed with SARS-CoV-2 infection. Medline, Embase databases and Google Scholar as well as relevant reviews were searched to identify appropriate studies from inception to 31st of August 2020. We calculated the pooled mean with 95% confidence intervals (95% CI) through a random-effect model meta-analysis. A total of 35 studies with 10,692 participants were considered for the review from which 23 studies with sufficient quantitative data were included in the meta-analysis. The pooled mean for liver enzymes and coagulation parameters did not significantly change in patients diagnosed with COVID-19 and remained within normal range. Notwithstanding potential bias from confounding factors in interpretation of data in this review, findings from the observational studies and case reports suggest that COVID-19 does not appear to have a significant impact on the transaminases or total bilirubin levels of patients with confirmed SARS-CoV-2 infection. Further controlled studies and larger sample size observational studies are needed with adequate reporting of other liver function parameters are warranted.

## Introduction

During December 2019, an outbreak of a novel coronavirus, the severe acute respiratory syndrome coronavirus 2 (SARS-CoV-2) was reported in the Wuhan City, China^1^. Initially, SARS-CoV-2 was reported as clusters of pneumonia with an unknown etiology^2^. Since then, the SARS-CoV-2 infection has rapidly and exponentially spread across the globe with majority of the infected cases reported first in Europe (Italy, Spain, UK) followed closely by the US and South America^3^. On 12th of March 2020, the World Health Organization (WHO) officially recognized and announced COVID-19 as a global pandemic disease. Globally by first week of September 2020, over 26 million people affected with SARS-CoV-2 infection and nearly 865,000 confirmed COVID-19 related deaths have been reported^4^. Unfortunately, no health care systems were prepared to combat this pandemic effectively resulting in major distress among all governments, health care professionals, researchers and communities worldwide^5^.

Available evidence suggest that SARS-CoV-2 primarily invades the upper respiratory system and progressively infects the lower respiratory tract^6^. This virus is thought to enter the respiratory system through infected droplets or mucosal contact by binding to the Angiotensin Converting Enzyme 2 (ACE2) receptor^7^. While some affected individuals remain asymptomatic^8^, majority of the individuals diagnosed with the SARS-CoV-2 infection experience fatigue, fever, dry cough as well as shortness of breath, nasal congestion and muscular pain^9^. Despite these symptoms, more than three-quarters of cases affected with SARS-CoV-2 resolve within 10–14 days of onset. However, the infection results in an estimated 3% mortality rate due to irreversible alveolar damage and progression to the fatal Acute Respiratory Distress Syndrome^10^. Furthermore, aged populations, individuals at high risk or those with cardiovascular comorbidities such as hypertension, diabetes, Chronic obstructive pulmonary disease and cancer develop a very severe form of the disease with significantly higher fatality rates^11^. At the time of our study there were no robust evidence for an effective treatment for COVID-19 other than preliminary data indicating dexamethasone therapy with ventilation support reduces mortality in hospitalized COVID-19 patients with severe respiratory complications^12^. Current preventive strategies to control spread of the SARS-CoV-2 infection include early diagnosis, quarantine and supportive treatment of infected patients^13^.

SARS-CoV is believed to be a systemic infection with multiorgan involvement including the heart, kidney, pancreas and liver. Nearly half of the SARS-CoV-2 infected individuals exhibit some degree of liver impairment which becomes more evident with the increasing severity of the disease^14^. In addition to this, RNA from the SARS-CoV-2 has been detected in blood and hepatic cells of the affected patients^15–17^. The high number of ACE2 receptors on the surface of the cholangiocytes in the liver^18,19^ bile duct cells^20^ presumably facilitate entry of SARS-COV-2 to the cells where they replicate leading to dysregulation of the liver function^21,22^. There is also evidence to suggest that most of the viruses which affect respiratory system are harmful to the liver cells through the CD8+ mediated immune response^23^.

Although data from a number of studies suggest an association between COVID-19 and abnormal liver function tests (LFTs) irrespective of health care setting^24–27^, however the evidence from recent epidemiological and clinical studies on LFTs in patients with SARS-CoV-2 infection is largely inconsistent and contradictory^24,28–31^. The prevalence of liver injury biomarker, alanine transferase (ALT) has been reported up to 32%, 38% and 39% in patients with COVID-19 from China, UK and USA, respectively^27,32,33^. On the other hand, some studies did not find any significant difference in ALT levels of COVID-19 patients based on the severity of the diseases^30,34^. Thus, as a result of reported inconsistency in findings for profile of liver chemistries in patients affected by COVID-19, a number of systematic reviews and meta-analyses have recently been published with a focus on the prevalence of abnormalities in liver biochemistry profile among patients affected by COVID-19 based on clinical severity and mortality of the disease^26,29,35–38^. A recent review of the evidence for the impact of COVID-19 on liver biochemistry profile concluded that despite reports on transient transaminase elevations, most laboratory changes in liver function test profile were mild-moderate and their clinical significance to COVID-19 related liver injuries remains unclear^39^. Another recent systematic review of available evidence on liver function tests found that although most of studies report significantly higher prevalence of liver test abnormalities in more severe hospitalized or non-surviving COVID-19 patients than milder cases, and given that other studies do not report significant changes in liver function tests of COVID-19 patients irrespective of severity, therefore in COVID-19 patients liver function abnormalities may not be a major characteristic^35^.

To our knowledge at the time of this publication, the reviews of the evidence for SARS-CoV-2 infection specific impact on the pooled estimate of liver functions is scarce. Therefore, we conducted a comprehensive systematic review with meta-analysis on the pooled mean of LFTs to provide an overview of the available evidence on the impact of SARS-CoV-2 infection on the liver function abnormalities.

## Methods

This SR and meta-analysis was followed and conducted according to the Preferred Reporting Items for Systematic reviews and Meta-analysis (PRISMA) reporting checklist (see Additional File 1)^40^.

### Search strategy

We searched Medline (via PubMed) and Embase databases without any language restrictions from inception to 31st of August, 2020. Key search terms related to COVID-19 and liver were used (see full list in Table 1). We also searched Google Scholar as well as performed manual searches of citations together with cross-checking of the references of recent reviews published until the 31st of August, 2020.

**Table 1:** Search strategies.

PubMed (Medline)	1. COVID-19.mp. [mp = title, abstract, original title, name of substance word, subject heading word, floating sub-heading word, keyword heading word, organism supplementary concept word, protocol supplementary concept word, rare disease supplementary concept word, unique identifier, synonyms] 2. Corona.mp. [mp = title, abstract, original title, name of substance word, subject heading word, floating sub-heading word, keyword heading word, organism supplementary concept word, protocol supplementary concept word, rare disease supplementary concept word, unique identifier, synonyms] 3. exp Coronavirus/ 4. 1 or 2 or 3 5. Liver Diseases/or Liver/or liver abnormalities.mp. 6. exp Liver Cirrhosis/or exp Liver Failure, Acute/or exp Acute-On-Chronic Liver Failure/or exp Liver Function Tests/or exp Liver Failure/ 7. 5 or 6 8. 4 and 7
EMBASE	1. COVID-19.mp. 2. Exp SARS coronavirus/ 3. Corona.mp. 4. Corona virus.mp. 5. 1 or 2 or 3 or 4 6. exp liver function test/or exp liver dysfunction/or exp liver enzymes or exp liver cell damage or exp liver function/ 7. 5 and 6

### Inclusion and exclusion criteria

The current analysis included all types of study designs, patients of any age or gender with a confirmed diagnosis of the COVID-19 disease. We excluded letters, editorials, reviews and commentaries. Outcomes of interest were LFTs and coagulation parameters reported for patients hospitalized with SARS-CoV-2 infection in any setting, those who had recovered or non-survivors. We assessed abnormalities in liver chemistry profiles of alanine aminotransferase (ALT), alkaline phosphatase (ALP), aspartate transaminase (AST), gamma-glutamyl transferase (GGT), total bilirubin, albumin, globulin and total protein. We also assessed changes in coagulation profiles of prothrombin time (PT) and activated partial thromboplastin time (aPTT). Studies that reported on patients with a history of liver disorders such as non-alcoholic fatty liver disease, acute/chronic liver injury, cirrhosis, liver failure, fibrosis and other liver related diseases were excluded. We also excluded non-English language studies due to lack of resources for accurate translation.

Two review authors independently assessed all identified studies for removal of duplication as well as initial eligibility assessment of title/abstract of all articles based on the eligibility criteria. Relevant studies were then subjected to full-text screening by the same reviewers. Disagreements were resolved by consensus or discussions with a third review author. The study characteristic and all outcomes of interest, including trial year, study setting and design, number of participants, hospitalization days and death-related information were extracted independently by two review authors.

### Data synthesis and statistical analysis

The data on the proportion of patients who had the abnormal (higher or lower) liver function tests were expressed as a percentage in narrative synthesis. The meta-analysis was performed using Review Manager Software. Case reports were excluded from the quantitative synthesis. The Mean and standard deviation (SD) were extracted from the included studies with the conversion of median and IQR to Mean and SD using the method described by Wan et al.^41^. The outcomes are presented as pooled mean together with the 95% confidence intervals (CI). Statistical heterogeneity of data was assessed using the I^2^ statistics with the random effect model applied when substantial heterogeneity was present (I^2^ > 50% or P ≤ 0.10)^42^. Subgroup analysis was planned where sufficient data from eligible studies was available. A funnel plot was used for visual inspection of publication bias^43^. A sensitivity analysis was performed by removing the studies with higher outliers to validate the robustness of the analyzed meta-data.

## Results

**Figure 1:**
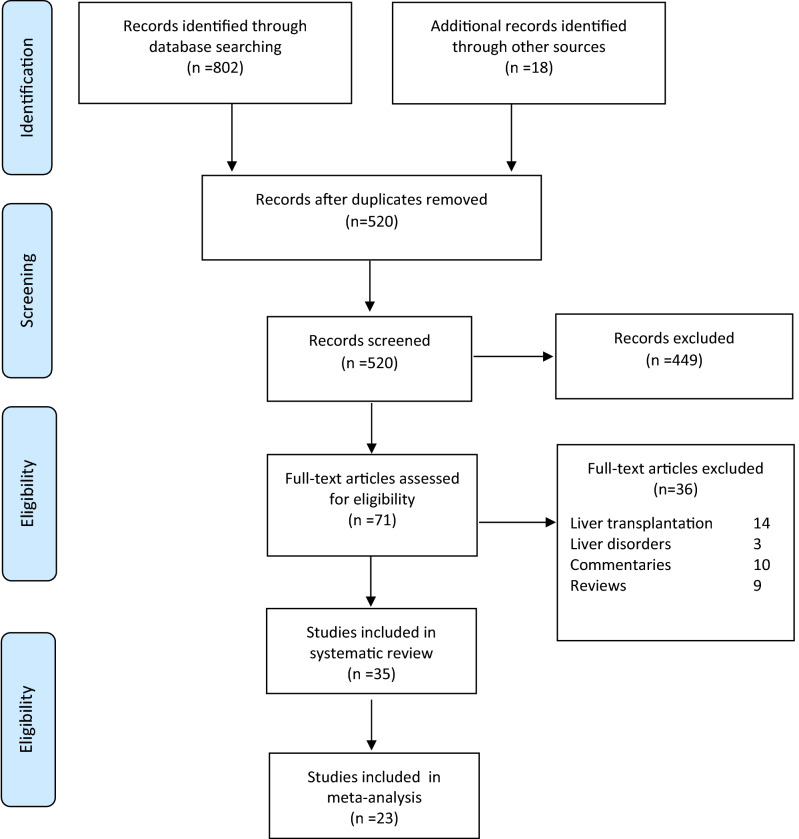
Study protocol according to PRISMA flow chart.

A total of 820 potential studies were identified through database and manual reference searches. After deduplication and screening a total of 35 studies were deemed eligible for this review with only 23 studies considered for the meta-analysis. A detailed study selection process based on PRISMA flow chart is presented in Fig. 1. The degree of agreement between review authors was 94% (33 studies agreed/35 studies) for inclusion of eligible studies and 92% for data extraction.

### Characteristics of the included studies

From the 35 included studies: retrospective studies (n = 25), case series (n = 6), case reports (n = 2) and cross sectional study (n = 2), majority of studies were from China (n = 31; 89%), followed by one study each from Singapore, USA, Germany and France^14,25,27,31,44–73^. The period of studies spanned from 11th of December 2019 to the 31st of August, 2020. The presence of SARS-COV-2 was confirmed using the molecular based technique of Reverse Transcriptase polymerase chain reaction (RT-PCR), while the diagnosis was based on the Chinese Center for Disease Prevention and Control (CDC) guidelines or WHO interim guidance. Table 2 summarizes the main characteristics of eligible studies.

**Table 2:** Characteristics of all included studies.

Study Author, Year	Country	Study design	Study duration	Diagnostic method	References
Cai et al. (2020)	China	Cross-sectional study	Jan 11 to Feb 21, 2020	RT-PCR and recommendations of CDC in China	^46^
Schattenberg et al. (2020)	Germany	Case series	March 3 and April 30, 2020	PCR positive for SARS-CoV-2 by throat swab	^72^
Meszaros et al. (2020)	France	Retrospective cohort study	March 10 to April 18, 2020	Reverse transcription polymerase chain reaction	^69^
Qi Wang et al. (2020)	China	Retrospective study	January 12, and March 17, 2020	New Coronavirus Pneumonia Diagnosis and Treatment Plan (Trial Edition 4–6)	^73^
Rui Hao et al. (2020)	China	Retrospective, cross-sectional study	January 17 to February 12, 2020	WHO interim guidance	^71^
Shasha Li et al. (2020)	China	Clinical trial	NA	Pneumonia Treatment Plan for the Novel Coronavirus Infection, National Health and Health Commission of the People’s Republic of China (Version 1–6)	^70^
Fan et al. (2020)	China	Retrospective study	January 20 through January 31, 2020	SARS-CoV-2 RNA by reverse transcriptase polymerase chain reaction	^74^
Cui et al. (2020)	China	Case report	17 days^a^	RT-PCR	^44^
Fan et al. (2020)	China	Retrospective, single-centre study	Jan 20 to 31, 2020	RT-PCR	^45^
Li et al. (2020)	China	Case series	Jan 14 to Feb 13, 2020	RT-PCR	^47^
Qain et al. (2020)	China	Retrospective study	Jan 20, to Feb 24, 2020	NR	^66^
Wang et al. (2020)	China	Retrospective study	Jan 1 to Feb 6, 2020	RT-PCR	^48^
Wu et al. (2020)	China	Retrospective study	Jan 22 to Feb 14, 2020	RT-PCR	^49^
Xie et al. (2020)	China	Retrospective study	Feb 2, to Feb 23, 2020	According to WHO interim guidance with laboratory-identified COVID-19	^27^
Yang et al. (2020)	China	Retrospective study	Jan 6 to Feb 25, 2020	RT-PCR and diagnosed by CDC China	^50^
Yang et al. (2020)	China	Single-centred, retrospective, observational study	Late-December 2019, to Jan 26, 2020	According to WHO interim guidance	^31^
Yao et al. (2020)	China	Retrospective study	NR	NR	^67^
Zhang et al. (2020)	China	Retrospective, single-centre study	Jan 8 to Feb 22, 2020	Commission of China	^52^
Guan et al. (2020)	China	Retrospective study	Dec 11, 2019, to Jan 29, 2020	By RT-PCR and diagnosis according to WHO interim guidance	^25^
Cai et al. (2020)	China	Retrospective study	Jan 11 to Feb 6, 2020	Based on WHO interim guidance	^53^
Xu et al. (2020)	China	Retrospective case series	Jan 10 to 26, 2020	By RT-PCR and diagnosis according to WHO interim guidance	^54^
Mo et al. (2020)	China	Retrospective single-centre study	Jan 1 to Feb 5, 2020	By RT-PCR	^55^
Wang et al. (2020)	China	Case series	Jan 16 to 29, 2020	By RT-PCR	^56^
Zhu et al. (2020)	China	Retrospective study	Jan 24 to Feb 20, 2020	Nucleic acid amplification	^58^
Wan et al. (2020)	China	Case Series	Jan 23 to Feb 8, 2020	By RT-PCR	^59^
Shi et al. (2020)	China	Retrospective study	Dec 20, 2019 to Jan 23, 2020,	Next-generation sequencing or RT-PCR	^60^
Huang et al. (2020)	China	Retrospective study	Dec 16, 2019 to Jan 2, 2020	Next-generation sequencing or RT-PCR	^61^
Xu et al. (2020)	China	Case report	Jan 21, 2020	RT-PCR	^62^
Lie et al. (2020)	China	Multicentre retrospective cohort	Dec 20, 2019 to March 8, 2020	RT-PCR	^75^
Wang et al. (2020)	China	Retrospective, single-centre case series	January 1 to Feb 3, 2020	WHO interim guidance	^64^
Yang et al. (2020)	China	Retrospective cohort study	January 17th to February 10th, 2020	By RT-PCR and diagnosis according to CDC China	^63^
Huang et al. (2020)	China	Retrospective study	December 2019 to January 2020	NR	^68^
Qian et al. (2020)	China	Retrospective study	20 January 2020 to 11 February 2020	RT-PCR	^65^
Young et al. (2020)	Singapore	Descriptive case series	Jan 23 to Feb 3, 2020	PCR	^51^
Arentz et al. (2020)	US	Case series	Feb 20 to March 5, 2020	By RT-PCR	^57^

*RT-PCR* real time polymerase chain reaction, *CDC* centre for disease control, *WHO* World Health Organization.

^a^The follow-up period.

### Characteristics of the included patients

The 35 studies included 10,692 patients diagnosed with SARS-CoV-2 of whom more than half consisted of females (n = 5469, 51.1%). The mean or median age of the patients ranged from 40 to 86 years. The most common comorbidities present in the included patients were diabetes, hypertension, kidney disease, acute cardiac injury, acute respiratory distress syndrome and shock. The major presenting symptoms of SARS-CoV-2 infection were fever, dry cough, expectoration, fatigue, anorexia, myalgia, dyspnea, pharyngalgia, diarrhea, nausea, breathlessness, chest tightness and dizziness. In addition to supportive and palliative care, the mainstay of treatment tailored to the presenting symptoms of the patients included antivirals, antibiotics, corticosteroids or immunoglobulin administration. The demographics, comorbidities, presenting symptoms and treatment modalities of patients diagnosed with COVID-19 are detailed in Table 3.

**Table 3:** Characteristics of participants in the included studies.

Study Author, Year	Sample size	Age (range)	Gender	Chronic diseases/comorbidities	Clinical severity n (%)	Signs and symptoms (n %)	Treatment n (%)
Cai et al. (2020)	417	47 years (33–59)^a^	Male: 81 (36%); Female: 336 (64%)	Diabetes: 12 (5%); Hypertension: 25 (11%); Liver disease: 4 (1.8%)	Mild: 326 (78.2); Severe: 91 (21.8)	Fever: 147 (65.3); Cough 73 (32.4)	NR
Qiu et al. (2020)	1	56 years	Female	Decompensated alcoholic cirrhosis with a history of gastric varices	Severe	Abdominal pain, fever and diarrhoea	Empiric antibiotic (Zosyn and Vancomycin), intravenous hydration
Schattenberg et al. (2020)	44	68 years (range 23–86)^a^	Male: 30 (68%); Female: 14 (32%);	NR	NR	Dyspnoea, fever and cough	Hydroxychloroquine, Protease inhibitor and Antibiotics
Meszaros et al. (2020)	244	67 ± 14 years	Male: 149 (63.7%); Female: 95 (36.3%);	Arterial hypertension, Cardiovascular disease, diabetes mellitus, chronic liver disease, Malignancy Immunosuppression and Chronic alcohol consumption	NR	NR	ACEI/ARB B-blockers, Diuretics, Calcium channel blocking agent
Qi Wang et al. (2020)	105	45 years (33.5–59.5)^a^	Male: 56 (53.3%); Female: 49 (46.7%);	Hypertension, Diabetes	Mild: 79 (48.1%); Severe: 26 (69.2%)	Fever and respiratory distress	Antipyretic, nutritional support, recombinant human interferon α-2b, lopinavir ritonavir tablet, reduced glutathione and compound glycyrrhizin
Shasha Li et al. (2020)	159	43 years	Male: 90 (56.6%); Female: 69 (44.4%);	Chronic hepatitis B virus, HBV-related cirrhosis, Hypertension, Diabetes, Coronary heart disease and fatty liver	Mild: 125 (78.6%); Severe: 34 (21.4%);	Fever	lopinavir/ritonavir and hydroxychloroquine
Fan et al. (2020)	148	50 years (36–64)^a^	Male: 73 (50.7%); Female: 75 (50.7%);	NR	Mild: 92 (62.2%); Severe: 10 (6.8%)	Fever 127 (85.8%) Cough 67 (45.3%) Expectoration 38 (26.7%) Diarrhea 6 (4.1%) Nausea and vomiting 3 (2.0%) Asymptomatic 5 (3.4%) With other liver diseases 9 (6.1%)	Antibiotics Antiviral Arbidol Oseltamivir Antipyretic analgesics
Cui et al. (2020)	1	55 days old	Female: 1	NR	NR	Rhinorrhoea and a dry cough	Inhaled interferon α-1b (15 μg, bid), amoxicillin potassium clavulanate (30 mg/kg, q8h, intravenous glucose tolerance test [IVGTT]), reduced glutathione, ursodeoxycholic acid, and traditional Chinese medicine lotus qingwen
Fan et al. (2020)	148	50 years^b^	Male 73 (49.3%); Female 75 (50.7%)	NR	NR	Fever 127 (85.8%); Cough 67 (45.3%); Expectoration 38 (26.7%); diarrhoea 6 (4.1%); Nausea and vomiting 3 (2.0%); Asymptomatic 5 (3.4%); other liver diseases 9 (6.1%)	Antibiotics: 50 (68%); Antiviral: 39 (50.8%); Arbidol: 13 (15.8%); Oseltamivir: 27 (36.5%); Antipyretic and analgesics: 14 (17.3%); No medication: 69 (94.3%)
Li et al. (2020)	25	73 years (55–100)^a^	Male: 10 (40%); Female: 15 (60%)	Hypertension: 16 (64%); Diabetes: 10 (40%); Heart diseases: 8 (32%); Kidney diseases: 5 (20%); Cerebral infarction: 4 (16%), Chronic obstructive pulmonary disease: 25 (8%); Malignant tumours: 2 (8%) and acute pancreatitis: 1 (4%)	NR	NR	NR
Qian et al. (2020)	324	51 years (15–88)^a^	Males: 167 (51.5%)	HBsAg positive: 20 (6.2%); Fatty Liver: 70 (21.6%)	NR	NR	NR
Wang et al. (2020)	339	69 years (65–76)^a^	Male: 166 (49%); Female: 173 (51%)	Hypertension: 138 (40.8%); Diabetes 54 (16.0%); Cardiovascular disease: 53 (15.7%); Cerebrovascular disease: 21 (6.2%); Chronic kidney disease: 13 (3.8%); Chronic liver disease: 2 (0.6%); COPD: 21 (6.2%); Malignancy: 15 (4.4%); Autoimmune disease: 5 (1.5%); Bacterial Infection 143 (42.8%); AKI 27 (8.1%); ARDS 71 (21.0%); Liver Enzyme Abnormalities 96 (28.7%); Acute cardiac injury 70 (21.0%); Arrhythmia 35 (10.4%); Cardiac insufficiency 58 (17.4%); Shock 8 (2.4%)	Moderate: 100 (29.5); Severe: 159 (46.9); Critical: 80 (23.6);	Fever 311 (92.0); Dry cough 179 (53.0); Expectoration 93 (27.5); Fatigue 135 (39.9); Anorexia 94 (27.8); Myalgia 16 (4.7); dyspnoea 138 (40.8); Pharyngalgia 13 (3.9); Diarrhoea 43 12.7); Nausea 13 (3.8); Chest tightness 88 (26.0); Dizziness 13 (3.8); Headache 12 (3.5);	NR
Wu et al. (2020)	80	46.10 ± 15.42^b^	Male: 39 (48.75%); Female: 41 (51.25%)	Cardiovascular and cerebrovascular diseases: 25 (31.25%); Endocrine system diseases: 5 (6.25%); Digestive system disease 3 (3.75%): Respiratory system diseases: 1 (1.25%): Malignant tumor: 1 (1.25%); Nervous system diseases: 1 (1.25%); Chronic kidney disease: 1 (1.25%); Chronic liver disease: 1 (1.25%)	NR		NR
Xie et al. (2020)	79	60 years (27–87)^a^	Male: 44 (55.7%); Female: 35 (44.3%)	Hypertension: 14 (17.7%); Diabetes mellitus: 8 (10.1%); CHD: 7 (8.9%)	NR	NR	NR
Yang et al. (2020)	92	69.8 ± 14.5^b^ 30–97^c^	Male 49/92 (53.3%); Female 43/92 (46.7%)	All 65/92 (70.7%); Hypertension 51/92 (56.1%); Heart disease 16/92 (20.7%); Diabetes 13/92 (18.3%); Cerebrovascular disease 10/92 (10.9%); Malignancy 4/92 (4.3%) Chronic liver disease 3/92 (3.3%); Chronic renal insufficiency 2/92 (2.2%); Haematological system disease 2/92 (2.2%); Chronic obstructive pulmonary disease 1/92 (1.1%)	NR	NR	NR
Yang et al. (2020)	52	59·7 ± 13·3^b^	Female: 17 (33%); Male: 35 (67%)	Chronic medical illness: 21 (40%); Chronic cardiac disease: 5 (10%); Chronic pulmonary disease: 4 (8%); Cerebrovascular disease: 7 (13·5%); Diabetes: 9 (17%); Malignancy: 2 (4%); Dementia: 1 (2%); Malnutrition: 1 (2%); Acute respiratory distress syndrome: 35 (67%); Acute kidney injury: 15 (29%); Cardiac injury: 12 (23%); Liver dysfunction: 15 (29%); Hyperglycaemia: 18 (35%); Gastrointestinal haemorrhage: 2 (4%); Pneumothorax: 1 (2%); Hospital-acquired pneumonia: 6 (11·5%); Bacteraemia: 1 (2%); Urinary tract infection: 1 (2%)	NR	Fever: 51 (98%); Cough: 40 (77%); Dyspnoea: 33 (63·5%); Myalgia: 6 (11·5%); Malaise: 18 (35%); Rhinorrhoea: 3 (6%); Arthralgia: 1 (2%); Chest pain: 1 (2%); Headache: 3 (6%); Vomiting: 2 (4%)	High flow nasal cannula: 33 (63·5%); Mechanical ventilation: 37 (71%); Non-invasive MV: 29 (56%); Invasive MV: 22 (42%); Prone position ventilation: 6 (11·5%); Extracorporeal membrane oxygenation: 6 (11·5%); Renal replacement therapy: 9 (17%); Vasoconstrictive agents: 18 (35%); Antiviral agents: 23 (44%); Antibacterial agents: 49 (94%); Glucocorticoids: 30 (58%); Immunoglobulin: 28 (54%)
Yao et al. (2020)	40	22–83^c^; 53.87 ± 15.84^b^	Male: 25 (62.5%); Female: 15 (37.5%)	NR	NR	NR	NR
Young et al. (2020)	18	47 years (31–73)^a^	Male: 9 (50%); Female: 9 (50%)	Any: 5 (28%)	NR	Fever: 13 (72); Cough: 15 (83); Shortness of breath: 2 (11); Rhinorrhoea: 1 (6); Sore throat: 11 (61); diarrhoea 3 (17)	Supplemental oxygen: 6 (33%); Admission to ICU: 2 (11%); Mechanical ventilation: 1 (6%); Antiviral: 5 (27.8)
Zhang et al. (2020)	115	49.52 ± 17.06^b^	Male: 49 (42.6%); Female: 66 (57.4%)	NR	Mild: 84 (73%); Severe: 31 (27%)	NR	NR
Guan et al. (2020)	1099	47.0 (35.0–58.0)^a^	Female: 459/1096 (41.9); Male: 637/1096 (58.1)	Any: 261 (23.7); Chronic obstructive pulmonary disease: 12 (1.1); Diabetes: 81 (7.4); Hypertension: 165 (15.0); Coronary heart disease: 27 (2.5); Cerebrovascular disease: 15 (1.4); Hepatitis B infection: 23 (2.1); Cancer: 10 (0.9); Chronic renal disease: 8 (0.7): Immunodeficiency: 2 (0.2)	NR	Fever: 473/1081 (43.8); Conjunctival congestion: 9 (0.8); Nasal congestion 53 (4.8); Headache: 150 (13.6); Cough: 745 (67.8); Sore throat: 153 (13.9); Sputum production: 370 (33.7); Fatigue: 419 (38.1); Haemoptysis: 10 (0.9); Shortness of breath: 205 (18.7); Nausea or vomiting: 55 (5.0); diarrhoea: 42 (3.8); Myalgia or arthralgia: 164 (14.9); Chills: 126 (11.5); Throat congestion: 19 (1.7); Tonsil swelling: 23 (2.1); Enlargement of lymph nodes: 2 (0.2); Rash: 2 (0.2)	NR
Cai et al. (2020)	298	47.5 (33–61)^a^	Male: 145 (48.66); Female: 155 (51.44)	T2DM: 18 (6.04); Hypertension: 47 (15.8); Cardiovascular diseases: 25 (8.39); Liver disease: 28 (9.4); Cancer: 4 (1.3)	Non-severe: 240 (80.5%); Severe: 58 (19.5%)	No symptoms: 30 (10.1); Fever: 218 (73.15); Cough: 105 (35.23); Fatigue: 13 (4.36); Headache: 5 (1.68); diarrhoea: 9 (3.02); Sore throat: 2 (0.67); Nasal congestion: 3 (1.01)	Lopinavir/ritonavir: 236 (79.2); NSAID: 121 (40.6); Corticosteroid: 91 (30.5); Gamma-globulin: 94 (31.5); Need ICU care: 30 (10.1); Invasive mechanical ventilation: 30 (10.1); Extracorporeal membrane oxygenation: 3 (1.0)
Xu et al. (2020)	62	41 (32–52)^a^	Male: 35 (56); Female 27 (44)	Any: 20 (32); Hypertension: 5 (8); Diabetes: 1 (2); Chronic obstructive pulmonary disease: 1 (2); Cerebrovascular disease: 1 (2); Renal diseases: 1 (2); Liver disease: 7 (11)	NR	Fever: 48 (77); Cough: 50 (81); Myalgia or fatigue: 32 (52); Expectoration: 35 (56); Haemoptysis: 2 (3); Headache: 21 (34); Diarrhoea 3 (8)	Antiviral treatment: 55 (89); Interferon alpha inhalation: 8 (13); Lopinavir/ritonavir: 4 (6); Arbidol + interferon alpha inhalation: 1 (2); Lopinavir/ritonavir + interferon alpha inhalation: 21 (34); Arbidol + lopinavir/ritonavir: 17 (28); Arbidol + lopinavir/ritonavir + interferon alpha inhalation: 4 (6); Antibiotics: 28 (45); Corticosteroid and gamma globulin: 16 (26)
Mo et al. (2020)	155	54 (42–66)^a^	Male 86	Hypertension: 37 (23.9), Diabetes: 15 (9.7), Cardiovascular diseases: 15 (9.7), Cerebrovascular diseases: 7 (4.5), Malignancy: 7 (4.5), Chronic liver diseases: 7 (4.5), Chronic renal diseases: 6 (3.9)	Stable: 63 (40.6); Serious: 55 (35.5); Critical: 37 (23.9)	Fever: 126 (81.3); Cough: 97 (62.6); Chest distress: 61 (39.4); Fatigue: 60 (73.2); Breath shortness: 50 (32.3); Myalgia or arthralgia: 50 (61.0); Anorexia: 26 (31.7)	Oxygen: 102 (65.8); Mechanical ventilation: 36 (23.2); Expectorant: 87 (56.1); Corticosteroid: 79 (51.0); Antiviral treatment: 45 (29.0); Arbidol: 31 (20.0); Lopinavir and ritonavir: 27 (17.4); Interferon inhalation: 30 (19.4); Immune enhancer: 14 (9.0); Thymalfasin: 11 (7.1); Immunoglobulin: 9 (5.8)
Wang et.al. (2020)	69	42 (35–62)^a^	Male: 32 (46%); Female 37 (54%)	Hypertension: 9 (13%); Cardiovascular disease: 8 (12%); Diabetes: 7 (10%); Malignancy: 4 (6%); Asthma: 2 (3%); Chronic hepatitis: 1 (1%)	NR	Sputum production 20 (29%); Dyspnoea 20 (29%); Oppression in chest 14 (20%); diarrhoea 10 (14%); Headache 10 (14%); Anorexia 7 (10%); Chest pain 6 (9%); Pharyngalgia 6 (9%); Dizziness 5 (7%); Palpitation 5 (7%); Vomiting 3 (4%); Cough 38 (55%); Fatigue 29 (42%); Myalgia 21 (30%)	Antiviral therapy: 66 (98.5%); Antibiotic therapy: 66 (98.5%); Antifungal therapy: 8 (11.9%); Use of corticosteroids: 10 (14.9%); Arbidol: 36 (53.7%)
Arentz et al. (2020)	21	70^b^; 43-92^c^	Male: 11 (52%); Female 10 (48%)	Asthma: 2 (9.1); Chronic obstructive pulmonary disease: 7 (33.3); Congestive heart failure: 9 (42.9); Diabetes: 7 (33.3); Rheumatologic disease: 1 (4.8); Obstructive sleep apnea: 6 (28.6); Chronic kidney disease: 10 (47.6); End-stage kidney disease: 2 (9.5); History of solid organ transplant: 2 (9.5); Cirrhosis 1 (4.8); Immunosuppression: (14.3)	Acute respiratory distress syndrome (ARDS): none: 1 (4.8); Mild: 2 (9.5); Moderate: 6 (28.6); Severe: 12 (57.1)	Cough:11 (47.6); Shortness of breath: 17 (76.2); Fever: 11 (52.4); Temperature (range) °C: 37.6 (35.3–39.2)	Use of non-invasive positive pressure ventilation: 4 (19.0): Use of high-flow oxygen therapy > 15 L/min: 1 (4.8): Required mechanical ventilation: 15 (71.0): Use of prone positioning for ARDS: 8 (50.0); Use of inhaled epoprostenol for ARDS: 5 (31.3); Use of vasopressors: 14 (67.0)
Zhu et al. (2020)	116	40 (27‐53)^a^	Male: 56 (46%); Female: 65 (54%)	Hypertension: 22 (19); Diabetes: 10 (9); Chronic obstructive pulmonary disease: 6 (5); Cerebrovascular disease: 5 (4); Mental disorder: 4 (3); Coronary heart disease: 5 (4); Tumour: 4 (3); Liver disease: 5 (4); Renal diseases: 2 (2)	NR	Fever: 84 (72); Cough: 73 (63); Myalgia or fatigue: 11 (9); Expectoration: 22 (19); Chest stuffiness: 5 (4); Haemoptysis: 1 (1); Headache: 3 (3); diarrhoea: 2 (2)	NR
Wan et al. (2020)	135	47 (36‐55)^a^	Male: 72 (53.3%); Female: 63 (46.7%)	Diabetes: 12 (8.9%); Cardiovascular disease: 7 (5.2%); Hypertension: 13 (9.6); Malignancy: 4 (3.0%); Pulmonary disease: 1 (0.7%); Chronic liver disease: 2 (1.5%); Acute respiratory distress syndrome: 21 (15.6%); Acute cardiac injury: 10 (7.4%); Acute kidney injury: 5 (3.7%); Secondary infection: 7 (17.5%); Shock: 1 (0.7%)	Severe: 40 (29.6%); mild: 95 (70.4%)	Fever: 120 (88.9%);Cough: 102 (76.5′%); Myalgia or fatigue: 44 (32.5%);Headache: 34 (32.5%); Pharyngalgia: 24 (17.7%); diarrhoea: 18 (13.3%); dyspnoea: 18 (13.3%); Chest tightness and shortness of breath:12 (8.8%); Sputum production: 12 (8.8%); Fear of cold: 14 (10.3%); Loss of appetite: 6 (4.4%); Palpitation: 5 (3.7%); Haemoptysis: 4 (3.0%); Retching: 4 (3.0%)	Antiviral therapy: 135 (100%); Antibiotic therapy: 59 (43.7%); Use of corticosteroid: 36 (26.7%); Traditional Chinese medicine: 124 (91.8%); Continuous renal replacement therapy: 5 (3.7%); Oxygen support: 90 (66.7%); Non-invasive ventilation or high‐flow nasal cannula: 34 (25.2%); Invasive mechanical ventilation: 1 (0.7%)
Shi et al. (2020)	81	49.5 (11.0)^a^	Male: 42 (52%), Female: 39 (48%)	Chronic pulmonary disease 9 (11%); Diabetes 10 (12%); Hypertension 12 (15%); Chronic renal failure 3 (4%); Cardiovascular disease 8 (10%); Cerebrovascular disease 6 (7%); Malignancy 4 (5%); Hepatitis or liver cirrhosis 7 (9%)	NR	Fever 59 (73%); Dyspnoea 34 (42%); Chest tightness 18 (22%); Cough 48 (59%); Sputum 15 (19%); Rhinorrhoea 21 (26%); Anorexia 1 (1%); Weakness 7 (9%); Vomiting 4 (5%); Headache 5 (6%); Dizziness 2 (2%); Diarrhoea 3 (4%)	NR
Huang et al. (2020)	41	49.0 (41.0–58.0)^a^	Male: 30 (73%); Female: 11 (27%)	Diabetes 8 (20%); Hypertension 6 (15%); Cardiovascular disease 6 (15%); Chronic obstructive pulmonary disease 1 (2%); Malignancy 1 (2%); Chronic liver disease 1 (2%)	NR	Fever 40 (98%); Cough 31 (76%); Myalgia or fatigue 18 (44%); Sputum production 11/39 (28%); Headache 3/38 (8%); Haemoptysis 2/39 (5%); Diarrhoea 1/38 (3%); Dyspnoea 22/40 (55%);	Antibiotic therapy 41 (100%); Antiviral therapy 38 (93%); Use of corticosteroid 9 (22%)
Xu et al. (2020)	1	50	Male	NR	NR	Fever, chills, cough, fatigue and shortness of breath	Interferon alfa-2b (5 million units twice daily, atomisation inhalation) and lopinavir plus ritonavir (500 mg twice daily, orally) as antiviral therapy, and moxifloxacin (0·4 g once daily, intravenously
Lie et al. (2020)	5771	56 (43–65)^a^	Male: 2,724 (47.2%)	NR	NR	NR	NR
Wang et al. (2020)	138	56 (42–68)^a^	Male: 75 (54.3%); Female: 63 (45.7%	Hypertension 43 (31.2); Cardiovascular disease 20 (14.5); Diabetes 14 (10.1); Malignancy 10 (7.2); Cerebrovascular disease 7 (5.1); COPD 4 (2.9); Chronic kidney disease 4 (2.9); Chronic liver disease 4 (2.9); HIV infection 2 (1.4),	NR	Fever 136 (98.6); Fatigue 96 (69.6); Dry cough 82 (59.4); Anorexia 55 (39.9); Myalgia 48 (34.8); dyspnoea 43 (31.2); Expectoration 37 (26.8); Pharyngalgia 24 (17.4); diarrhoea 14 (10.1); Nausea 14 (10.1); Dizziness 13 (9.4); Headache 9 (6.5); Vomiting 5 (3.6); Abdominal pain 3 (2.2)	Antiviral therapy 124 (89.9); Glucocorticoid therapy 62 (44.9); invasive mechanical ventilation 17 (12.32); extracorporeal membrane oxygenation 4 (2.9)
Yang et al. (2020)	149	45.11 ± 13.35^b^	Male: 81; Female: 68	Cardio-cerebrovascular disease 28 (18.79%); Digestive system diseases 8 (5.37%); Endocrine diseases 9 (6.04%); Malignant tumour 2 (1.34%); Neural system diseases 0 (0%); Respiratory system diseases 1 (0.67%); Others 4 (2.68%)	NR	Fever 114 (76.51%); cough 87 (58.39%); Expectoration 48 (32.21%); dyspnoea 2 (1.34%); Muscle pain 5 (3.36%); Headache 13 (8.72%); Sore throat 21 (14.09%); Snotty 5 (3.36%); Chest pain 5 (3.36%); Chest tightness 16 (10.74%); Chill 21 (14.09%); diarrhoea 11 (7.38%); Nausea and vomiting 2 (1.34%)	Antibiotic treatment 34 (22.82%); Antifungal treatment 0 (0.0%); Antiviral treatment 140 (93.96%); Interferon administration 144 (96.64%); Glucocorticoids 5 (3.36%); Immunoglobulin therapy 19 (12.75%)
Huang et al. (2020)	34	56.24 ± 17.14^b^	Male: 14 (41.2%); Female: 20 (58.8%)	Diabetes 4 (11.8%); Hypertension: 8 (23.5%); Cardiovascular disease: 6 (17.6%); Chronic obstructive: 1 (2.9%); Pulmonary disease: 2 (5.9%); Malignancy: 3 (8.8%); Chronic liver disease: 1 (2.9%); Hyperuricemia: 1 (2.9%); Hypothyroidism: 2 (5.9%); HIV infection: 2 (5.9%)	NR	Fever: 32 (94.1%); Cough: 17 (50.0%); Myalgia or fatigue: 22 (64.7%); Sputum production: 8 (23.5%); Headache: 2 (5.9%); diarrhoea: 5 (14.7%); dyspnoea: 5 (14.7%)	Antibiotic therapy: 31 (91.2%); Antiviral therapy (other drugs but not lopinavir/ritonavir): 32 (94.1%); Antiviral therapy (switch to lopinavir/ritonavir later): 9 (26.5%); Use of corticosteroid 21 (61.8%)
Qian et al. (2020)	91	50 (36.5–57)^a^	Female: 54 (59.34%); Male: 37 (40.66%)	Hypertension: 15 (16.48%); Diabetes Mellitus: 8 (8.79%); Cardiovascular and cerebrovascular disease: 3 (3.30%)	NR	Fever 65 (71.43); unknown 2 (2.2); cough 55 (60.44%); Fatigue 40 (43.96%); Expectoration 30 (32.97%); Anorexia 23 (25.27%); diarrhoea 21 (23.08%); Chest distress: 17 (18.68%); Nausea: 11 (12.09%); Shortness of breath: 10 (10.99%); dyspnoea: 3 (3.3%); Headache7 (7.69%); Vomiting 6 (6.59%); Myalgia: 5 (5.49%); Back discomfort 3 (3.3%)	NR

*T2DM* type 2 diabetes mellitus, *ACEi/ARBi* Angiotensin II Receptor Blockers and angiotensin-converting enzyme inhibitor, *NR* not reported.

^a^The Median with IQR.

^b^Mean with SD.

^c^The range.

### Abnormalities of the plasma proteins

#### Albumin

**Figure 2:**
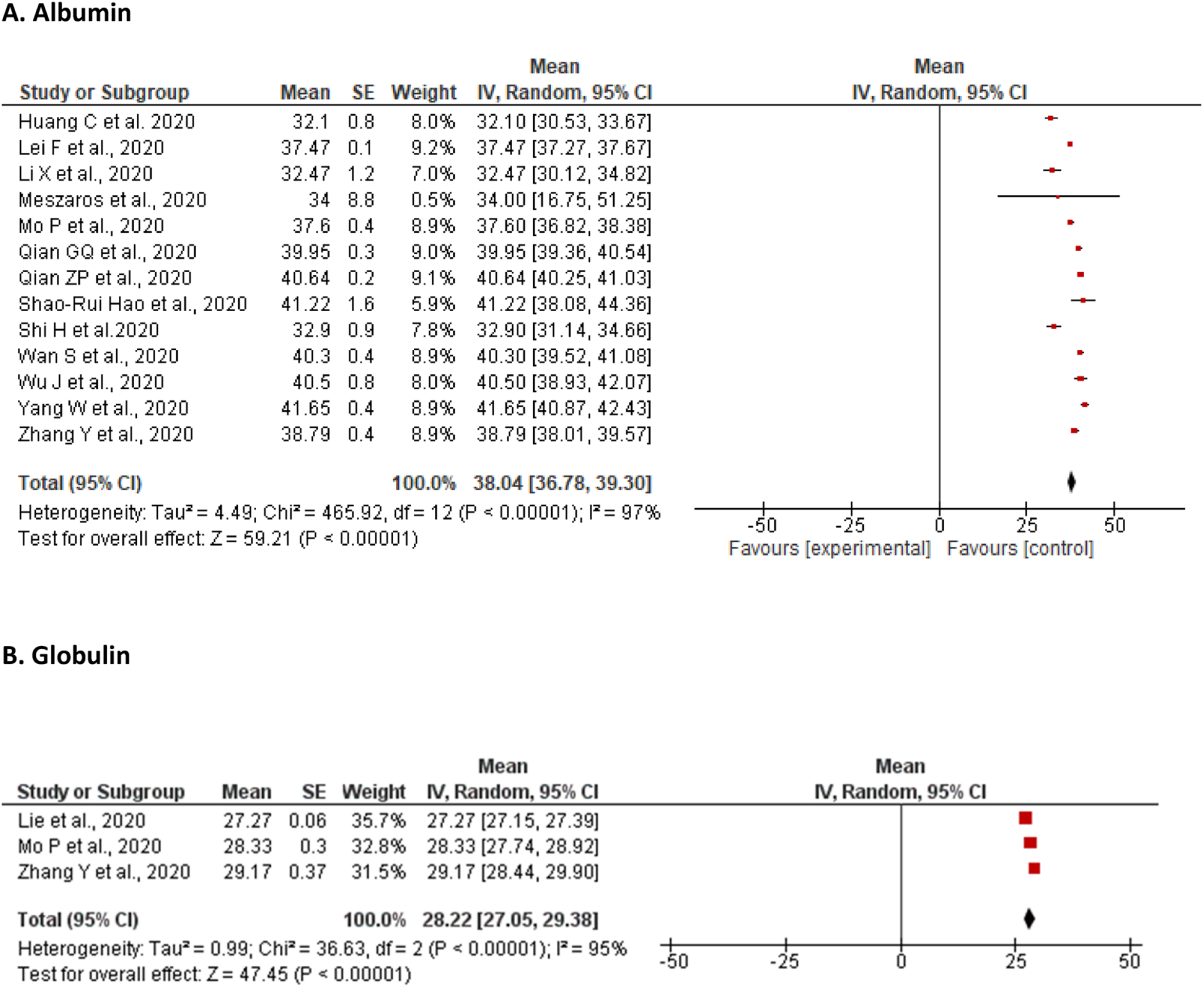

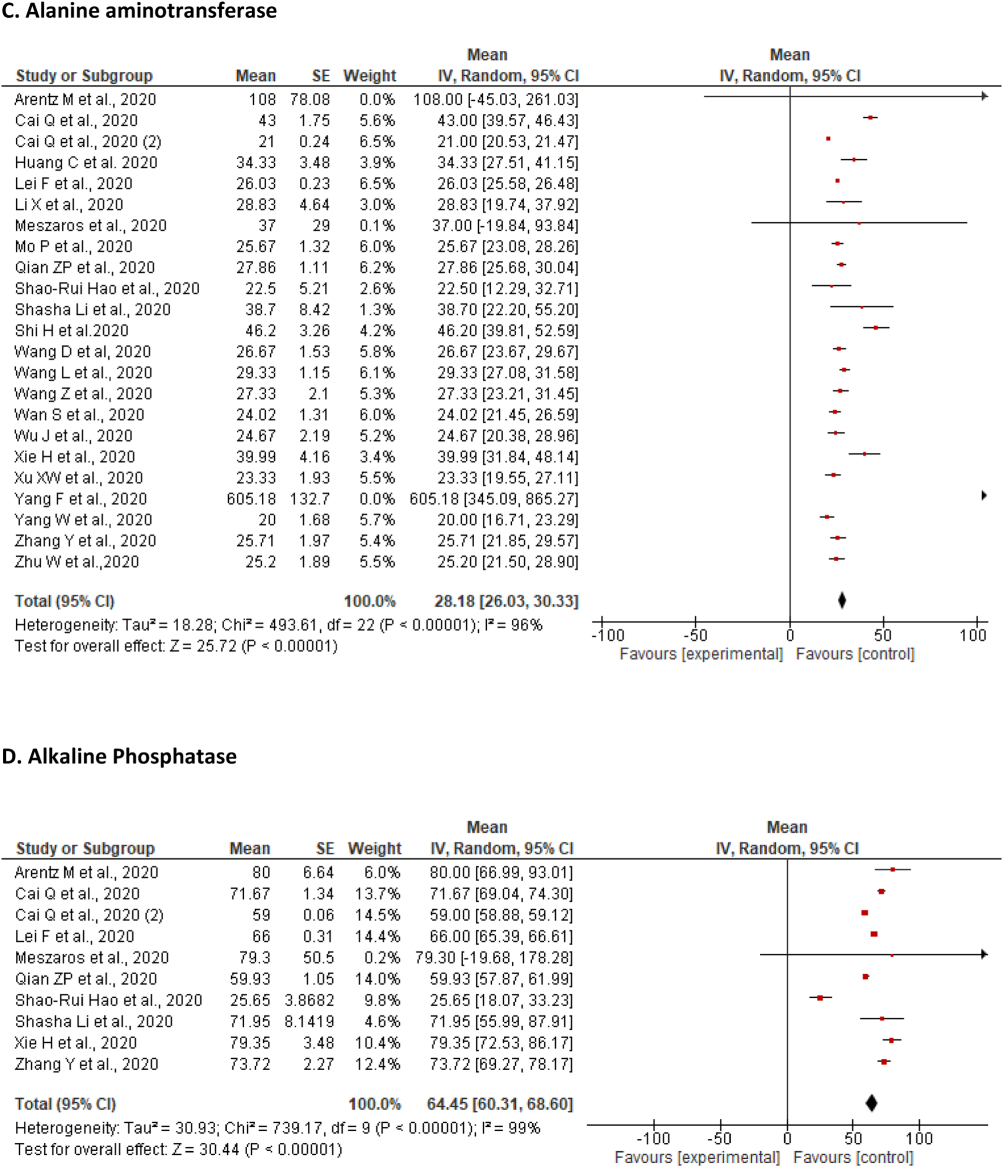

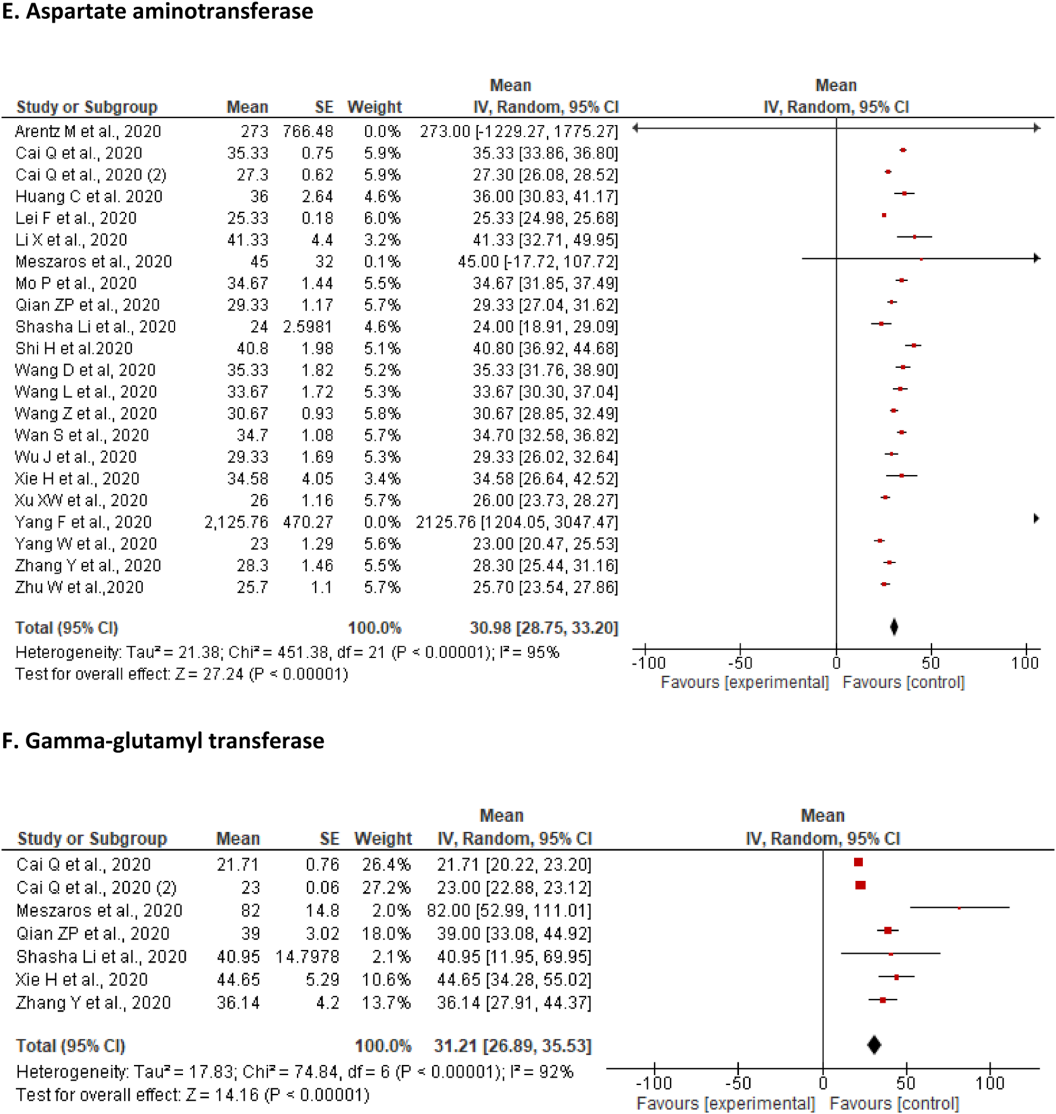
Meta-analyses of the pooled mean of Liver enzymes. (**A**) Albumin; (**B**) Globulin; (**C**) Alanine aminotransferase; (**D**) Alkaline phosphatase; (**E**) Aspartate aminotransferase; (**F**) Gamma-glutamyl transferase.

A meta-analysis of 13 studies revealed that the pooled mean of albumin levels was 38.04 g/L [36.78, 39.30] with a significant level of heterogeneity (I^2^ = 97%, P < 0.00001). Majority of the studies did not report the number of patients who showed changes in albumin levels. However, Yang et al. and Huang et al. reported elevation of albumin in 2% (n = 3) and 20.6% (n = 7) of affected patients, respectively (Fig. 2A). In contrast, lower level of albumin were reported widely ranging from 6% (n = 9)^63^ to 100% (n = 5)^47^.

#### Globulin

The pooled mean of globulin level from three studies was found to be 28.22 g/L [27.05, 29.38] with a significant level of heterogeneity (I^2^ = 95%, P < 0.00001). Only Zhang et al. reported an elevation of globulin in a third of the included patients (n = 42; 36.5%) (Fig. 2B).

#### Total protein

Total protein was only reported by Xu et al. and estimated to be 56 g/L in the patients with SARS-CoV-2 infection.

#### Alanine aminotransferase (ALT)

A total of 23 studies reported on changes in ALT levels of the included patients. The pooled mean of ALT was found to be 28.18 U/L [26.03, 30.33] with a significant level of heterogeneity (I^2^ = 96%, P < 0.00001). The proportion of patients with an elevated ALT level ranged from 3.75% (n = 3)^49^ to 36.79% (n = 117)^48,50,53,64^, while a decrease from the lower limit of ALT levels varied from 2 (1.34%) to 67% (n = 46)^56^ (Fig. 2C).

A sensitivity analysis performed by removing two studies^50,57^ with outlier ALT values did not show a noticeable change in the pooled results revealing mean of 28.65 U/L [26.47, 30.83] with a significant heterogeneity (I^2^ = 92%, P < 0.00001) (Additional File 2, Supplementary Data Fig. S1).

#### Alkaline phosphatase (ALP)

A total of ten studies reported on changes in levels of ALP of the included patients. The pooled mean for ALP was found to be 64.45 U/L [60.31, 68.60] with a significant level heterogeneity (I^2^ = 99%, P < 0.00001). The proportion of patients with an elevated ALP levels ranged from 0.3% (n = 1) to 6.6% (n = 21)^46,53^, while a decrease from the lower limit of ALP levels was not reported by any study (Fig. 2D).

#### Aspartate aminotransferase (AST)

A total of 22 studies reported on the changes in AST levels of the included patients. The pooled mean of AST was found to be 30.98 U/L [28.75, 33.20] with a significant level of heterogeneity (I^2^ = 95%, P < 0.00001). The proportion of patients with an elevation of AST ranged from 3.75% (n = 3)^49^ to 36% (n = 9)^47^. In contrast, a decrease from the lower limit of AST level (n = 50; 72%) was reported only by Wang et al. (Fig. 2E).

The sensitivity analysis performed by removing two studies with outliers did not alter the findings of original analysis of the mean 32.01 U/L [29.61, 34.42] with the heterogeneity of I^2^ = 96%; P < 0.00001 (Additional File 2, Supplementary Fig. S2).

#### Total bilirubin (TB)

A total of 17 studies reported on the changes in levels of TB of the included patients. The pooled mean of TB was found to be 11.36 μmol/L [10.35, 12.38] with a significant level of heterogeneity (I^2^ = 96%, P < 0.00001). The proportion of patients with an elevated TB level ranged from 1.25% (n = 1)^49^ to 44.02% (n = 140)^46^. Whereas, only Yang et al., 2020 reported a decrease in TB levels among seven patients (4.70%) (Supplementary Fig. S3).

A sensitivity analysis by removing two studies demonstrated no noticeable change in the overall results (11.25 μmol/L [10.27, 12.22]) with the heterogeneity (I^2^ = 96%, P < 0.00001) (Additional File 2, Supplementary Data Fig. S4).

#### Gamma-glutamyl transferase (GGT)

A total of seven studies reported on the levels of GGT in the included patients. The pooled mean of GGT was found to be 31.21 U/L [26.89, 35.53] with a significant level of heterogeneity (I^2^ = 96%, P < 0.00001). The proportion of patients with an elevated level of GGT ranged from 0.9% (n = 3)^66^ to 28.61% (n = 91)^53^. However, none of the studies reported a decreased level of GGT among the affected patients (Fig. 2F).

#### Prothrombin time (PtT)

**Figure 3:**
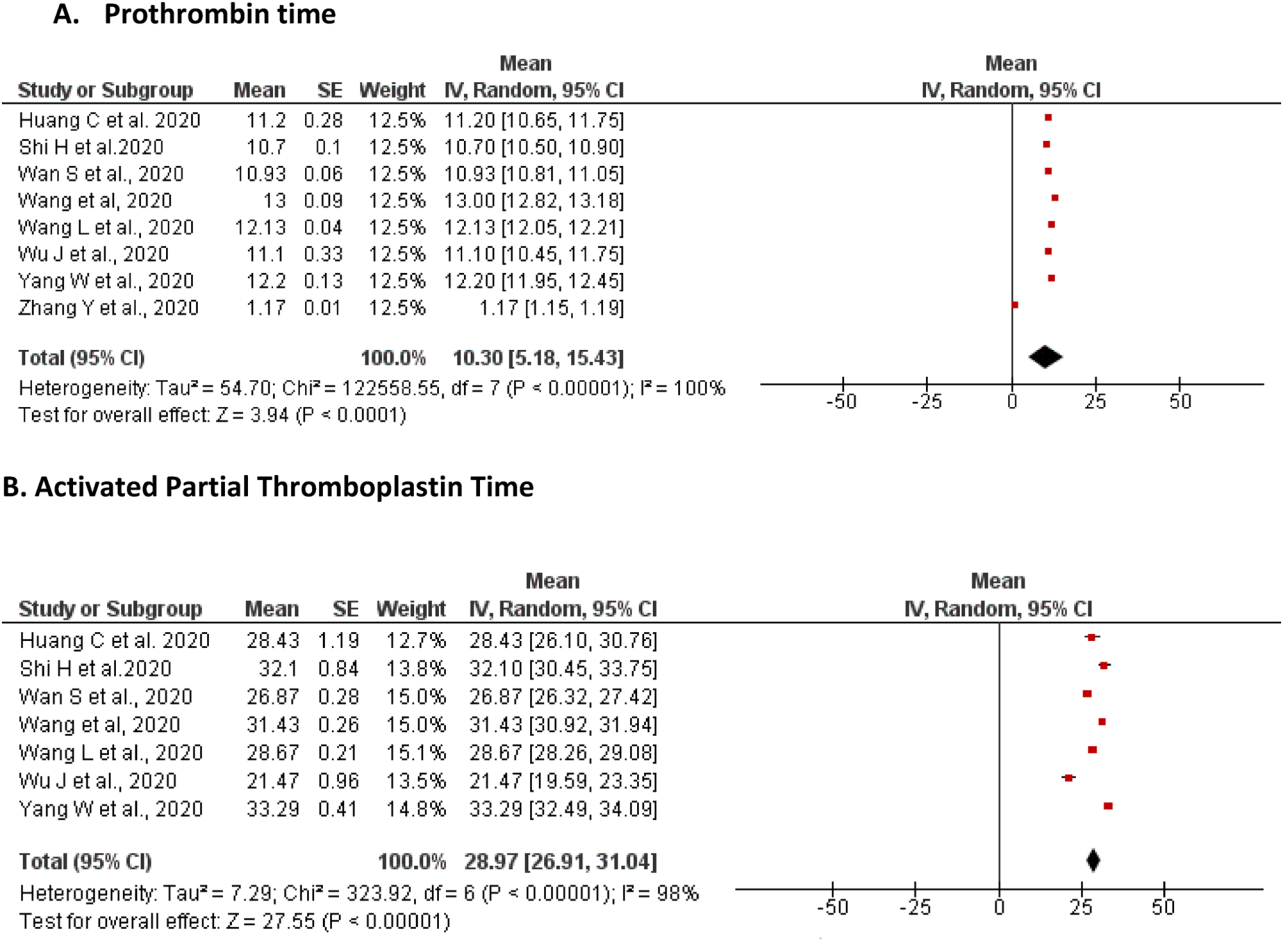
Meta-analyses of the pooled mean of coagulation parameters. (**A**) prothrombin time; (**B**) Activated Partial Thromboplastin time.

A total of eight studies reported on changes in the levels of PtT of the included patients. The pooled mean of PtT was found to be 10.30 s [5.18, 15.43] with a significant level of heterogeneity (I^2^ = 100%, P < 0.00001). An elevation of PtT was reported by Zhang et al. and Yang et al. among 52.2% (n = 60) and 11.41% (n = 17) of the patients, respectively. In contrast, a decrease from the lower limit in PtT levels was reported by Yao et al. (7.5%, n = 3), Wu et al. (3.75%, n = 3) and Yang et al. (2.68%, n = 4) (Fig. 3A).

#### Activated partial thromboplastin time (aPTT)

A total of seven studies reported on the levels of aPTT in the included patients. The pooled mean of aPTT was found to be 28.97 s [26.91, 31.04] with a significant level of heterogeneity (I^2^ = 100%, P < 0.00001). An elevation of aPTT was reported by Yang et al., among quarter of the patients (n = 40; 26.85%). In contrast, a decrease from the lower limit of aPTT level was reported by Wu et al., in only two patients (Fig. 3B).

#### Publication bias

The visual inspection of the funnel revealed that all studies concentrated on a single point which could represent a potential publication bias in the studies.

## Discussion

We have shown in this systematic review with meta-analysis that most of the liver enzymes and coagulation parameters in patients diagnosed with SARS-CoV-2 infection are not significantly impacted by COVID-19. We have presented an in depth analysis of pooled mean data for liver chemistries and each of the liver function test markers in patients diagnosed with SARS-CoV-2 infection upon hospital admission. The same findings were observed with the prothrombin and activated prothrombin time. In these patients, the enzymatic liver function tests for albumin, globulin, ALT, ALP, AST, GGT and TB together with the coagulation profile were not significantly associated with COVID-19 at initial presentation.

In this review, all the mean values of liver function tests were found to be within the normal range and not associated with disease progression consistent with the findings of Wang et al.^38^ and Mantovani et al.^31^ meta-analyses. Although Wang et al. conducted a meta-analysis on gastrointestinal symptoms in patients with COVID-19, however they only reviewed evidence of liver injury qualitatively. Their review of evidence on liver injury in patients with COVID-19 revealed that 2.6–53% of the affected patients had abnormal levels of ALT, AST and TB based on the severity of the disease^38^. A recent meta-analysis of observational studies on the prevalence of liver injuries in patients with SARS-CoV-2 infection further showed a relatively low association at initial presentation but reported important changes in liver enzymes of the patients with severe form of the disease^37^. However, similar to the systematic review by Wang et al.^38^, the study by Mantovani et al.^31^ did not include a meta-analysis of liver function tests whereas we analysed pooled means of liver enzymes as well as the profiles of coagulative and fibrinolytic pathways in patients with COVID-19.

A meta-analysis conducted by Mao et al. on the prevalence of abnormal liver enzyme levels in confirmed COVID-19 patients, reported the pooled prevalence of elevated ALT, AST, and TB among patients with COVID-19 as 18%, 21% and 6% respectively^26^. Our meta-analysis also revealed reduced ALT and albumin levels in patients with COVID-19 albeit with wide ranges. However, only one study reported reduced AST and TB levels (72% and 4.70%, respectively)^56^. Although we did not find any increase in the coagulation time, nevertheless the physicians need to be vigilant and closely monitor the coagulation profile of the patients with COVID-19. Additionally, we found elevated levels of GGT, PtT, and ALP in several studies. Of note, none of the studies evaluated the proportion and mean changes in total protein.

The underlying mechanism of liver injury in patients with SARS-CoV-2 infection is poorly understood^35^. Several potential mechanisms have been proposed. There is evidence to suggest that liver functional impairment in the COVID-19 patients could have resulted from drug-induced hepatotoxicity^76^. Another mechanism for liver injury involves the inflammatory response of immune system, especially cytokine storms which can lead to the damage of the liver cells^77^. While contribution from other viral infections or drug-induced toxicity cannot be ruled out, given the propensity of data on abnormal liver tests in severe cases of COVID-19 it is plausible that liver injury may also occur from the direct effects of SARS-CoV-2 as part of a complex multifactorial mechanism but further focused research is needed to clarify the contribution of abnormal liver tests to the pathogenetic mechanism of COVID-19^24,35,36^.

## Limitations

There are several limitations and confounding factors that my present as potential bias and therefore results should be interpreted with caution. The major imitation of this review was the presence of a high level of heterogeneity across all included studies. We could not assess sources of heterogeneity in our included studies, due to lack of reporting of subgroup data such as comorbidities, hospitalization (ICU setting) and asymptomatic patients. There was also a significant publication bias noted in this meta-analysis. Moreover, we did not conduct quality assessment of included studies due to the low number of studies reporting assessment of liver function tests as the primary outcome, hence assessing the quality of assessment was deemed not appropriate. In addition, most studies were retrospective with small sample sizes and we only identified one controlled trial.

Since COVID-19 is a rapidly evolving disease and given majority of our included studies were from China, generalizability of these results are not possible and therefore ethnic subgroup analysis and epigenetics were not possible. One of the main drawbacks of the currently available literatures on COVID-19 is the lack of control group reporting in studies. Hence, there is a growing need to compare the laboratory liver function tests conducted in the primary studies with a comparator or a control group. Another limitation is the lack of sufficient data on the proportion of patients with changes in liver function parameters at discharge. Furthermore, most studies did not report on some of the variables that we investigated including globulin, GGT, total protein and coagulation profile. Therefore, to assess whether there is a causal association between SARS-CoV-2 infection and liver injury, it is important that future studies report other important liver function tests, the proportion of patients with altered levels of liver function parameters and number of patients who have recovered from COVID-19 at discharge.

## Conclusion

The findings from the available evidence to date from observational studies and case reports indicate that at least the transaminases and total bilirubin levels in COVD-19 diagnosed patients appear not to have significantly changed. Future studies would benefit from inclusion of a control group and larger sample size observational studies are needed with reporting of the number of patients with changes in levels of liver function abnormalities.

## Supplementary Information

Supplementary Information 1.

Supplementary Information 2.

## Data Availability

The datasets used and/or analysed during the current study are available from the corresponding author on reasonable request.

## References

[1] Wu D, Wu T, Liu Q, Yang Z (2020). The SARS-CoV-2 outbreak: What we know. Int. J. Infect. Diseases IJID Off. Publ. Int. Soc. Infect. Diseases.

[2] Sun J (2020). COVID-19: Epidemiology, evolution, and cross-disciplinary perspectives. Trends Mol. Med..

[3] Adhikari SP (2020). Epidemiology, causes, clinical manifestation and diagnosis, prevention and control of coronavirus disease (COVID-19) during the early outbreak period: A scoping review. Infect. Dis. Poverty.

[4] World Health Organization. Coronavirus disease (COVID-19): Weekly epidemiological update, 11 October 2020. (2020).

[5] Kannan S, Ali PSS, Sheeza A, Hemalatha K (2020). COVID-19 (novel coronavirus 2019—Recent trends. Eur. Rev. Med. Pharmacol. Sci..

[6] Tian S (2020). Characteristics of COVID-19 infection in Beijing. J. Infect..

[7] Hoffmann M (2020). SARS-CoV-2 cell entry depends on ACE2 and TMPRSS2 and is blocked by a clinically proven protease inhibitor. Cell.

[8] Singhal T (2020). A review of coronavirus disease-2019 (COVID-19). Indian J. Pediatr..

[9] Jin, X. *et al.* Epidemiological, clinical and virological characteristics of 74 cases of coronavirus-infected disease 2019 (COVID-19) with gastrointestinal symptoms. Gut. **69**(6), 1002–1009. 10.1136/gutjnl-2020-320926 (2020). 10.1136/gutjnl-2020-320926 PMC7133387 32213556

[10] Jiang F (2020). Review of the clinical characteristics of coronavirus disease 2019 (COVID-19). J. Gen. Intern. Med..

[11] Guo, W. *et al.* Diabetes is a risk factor for the progression and prognosis of COVID-19. *Diabetes Metab Res Rev.*10.1002/dmrr.3319 (2020). 10.1002/dmrr.3319 PMC7228407 32233013

[12] Horby, P. *et al.* Dexamethasone in hospitalized patients with COVID-19-preliminary report. *N. Engl. J. Med.* **383**(21), 2030–2040 10.1056/NEJMoa2022926. (2020). 10.1056/NEJMoa2022926 PMC7556338 33031652

[13] Pascarella, G. & Strumia, A. COVID-19 diagnosis and management: a comprehensive review. *J Intern Med.* **288**(2), 192–206 10.1111/joim.13091 (2020). 10.1111/joim.13091 PMC7267177 32348588

[14] Qi X (2020). Multicenter analysis of clinical characteristics and outcome of COVID-19 patients with liver injury. J. Hepatol..

[15] Zhang C, Shi L, Wang FS (2020). Liver injury in COVID-19: Management and challenges. Lancet Gastroenterol. Hepatol..

[16] Feng G (2020). COVID-19 and liver dysfunction: Current insights and emergent therapeutic strategies. J. Clin. Transl. Hepatol..

[17] Li MY, Li L, Zhang Y, Wang XS (2020). Expression of the SARS-CoV-2 cell receptor gene ACE2 in a wide variety of human tissues. Infect. Dis. Poverty.

[18] Shang, J. *et al.*Cell entry mechanisms of SARS-CoV-2. Proc Natl Acad Sci USA. 2020 May 26;117(21):11727-11734 10.1073/pnas.2003138117 (2020). 10.1073/pnas.2003138117 PMC7260975 32376634

[19] Chai, X. *et al.* Specific ACE2 expression in cholangiocytes may cause liver damage after 2019-nCoV infection. *BioRxiv* (2020). 10.1101/2020.02.03.931766

[20] Hamming I (2004). Tissue distribution of ACE2 protein, the functional receptor for SARS coronavirus. A first step in understanding SARS pathogenesis. J. Pathol..

[21] Zhang Y (2020). New understanding of the damage of SARS-CoV-2 infection outside the respiratory system. Biomed. Pharmacother..

[22] Xu L, Liu J, Lu M, Yang D, Zheng X (2020). Liver injury during highly pathogenic human coronavirus infections. Liver Int..

[23] Adams DH, Hubscher SG (2006). Systemic viral infections and collateral damage in the liver. Am. J. Pathol..

[24] Bertolini A (2020). Abnormal liver function tests in patients with COVID-19: Relevance and potential pathogenesis. Hepatology (Baltimore, MD).

[25] Guan WJ (2020). Clinical characteristics of coronavirus disease 2019 in China. N. Engl. J. Med..

[26] Mao R (2020). Manifestations and prognosis of gastrointestinal and liver involvement in patients with COVID-19: A systematic review and meta-analysis. Lancet Gastroenterol. Hepatol..

[27] Xie, H., Zhao, J. & Lian, N. Clinical characteristics of non-ICU hospitalized patients with coronavirus disease 2019 and liver injury: A retrospective study. Liver Int. 2020 Jun;40(6):1321–1326. 10.1111/liv.14449 (2020). 10.1111/liv.14449 PMC7228333 32239591

[28] Kulkarni AV (2020). Systematic review with meta-analysis: Liver manifestations and outcomes in COVID-19. Aliment. Pharmacol. Ther..

[29] Kukla M (2020). COVID-19, MERS and SARS with concomitant liver injury—Systematic review of the existing literature. J. Clin. Med..

[30] Li X (2020). Risk factors for severity and mortality in adult COVID-19 inpatients in Wuhan. J. Allergy Clin. Immunol..

[31] Yang X (2020). Clinical course and outcomes of critically ill patients with SARS-CoV-2 pneumonia in Wuhan, China: A single-centered, retrospective, observational study. Lancet Respir. Med..

[32] Lok, J. & Gess, M. Liver dysfunction in COVID-19: A useful prognostic marker of severe disease? *Frontline Gastroenterology* (2020). 10.1136/flgastro-2020-101689 10.1136/flgastro-2020-101689 PMC8231420 34249314

[33] Richardson S (2020). Presenting characteristics, comorbidities, and outcomes among 5700 patients hospitalized with COVID-19 in the New York City area. JAMA.

[34] Wan S (2020). Clinical features and treatment of COVID-19 patients in northeast Chongqing. J. Med. Virol..

[35] Ali N, Hossain K (2020). Liver injury in severe COVID-19 infection: Current insights and challenges. Expert Rev. Gastroenterol. Hepatol..

[36] Ali N (2020). Relationship between COVID-19 infection and liver injury: A review of recent data. Front. Med..

[37] Mantovani, A., Beatrice, G. & Dalbeni, A. Coronavirus disease 2019 and prevalence of chronic liver disease: A meta-analysis. Liver Int. 2020 Jun;40(6):1316-1320. 10.1111/liv.14465 (2020). 10.1111/liv.14465 32329563

[38] Wang H, Qiu P, Liu J, Wang F, Zhao Q (2020). The liver injury and gastrointestinal symptoms in patients with Coronavirus Disease 19: A systematic review and meta-analysis. Clin. Res. Hepatol. Gastroenterol..

[39] Garrido I, Liberal R, Macedo G (2020). COVID-19 and liver disease—What we know on 1st May 2020. Aliment. Pharmacol. Ther..

[40] Moher D, Liberati A, Tetzlaff J, Altman DG, Group, P (2009). Preferred reporting items for systematic reviews and meta-analyses: The PRISMA statement. PLoS Med..

[41] Wan X, Wang W, Liu J, Tong T (2014). Estimating the sample mean and standard deviation from the sample size, median, range and/or interquartile range. BMC Med. Res. Methodol..

[42] Delgado-Rodríguez M, Sillero-Arenas M (2018). Systematic review and meta-analysis. Med. Intensiva.

[43] Egger M, Smith GD, Schneider M, Minder C (1997). Bias in meta-analysis detected by a simple, graphical test. BMJ (Clinical Research Ed.).

[44] Cui Y (2020). A 55-day-old female infant infected with 2019 novel coronavirus disease: Presenting with pneumonia, liver injury, and heart damage. J. Infect. Dis..

[45] Fan, Z. *et al.* Clinical features of COVID-19-related liver damage. *Clin. Gastroenterol. Hepatol.* **18**(7), 1561-1566. 10.1016/j.cgh.2020.04.002 (2020). 10.1016/j.cgh.2020.04.002 PMC7194865 32283325

[46] Cai Q (2020). COVID-19: Abnormal liver function tests. J. Hepatol..

[47] Li X (2020). Clinical characteristics of 25 death cases with COVID-19: A retrospective review of medical records in a single medical center, Wuhan, China. Int. J. Infect. Diseases IJID Off. Publ. Int. Soc. Infect. Diseases.

[48] Wang L (2020). Coronavirus disease 2019 in elderly patients: Characteristics and prognostic factors based on 4-week follow-up. J. Infect..

[49] Wu J (2020). Clinical characteristics of imported cases of COVID-19 in Jiangsu Province: A multicenter descriptive study. Clin. Infect. Diseases Off. Publ. Infect. Diseases Soc. Am..

[50] Yang F (2020). Analysis of 92 deceased patients with COVID-19. J. Med. Virol..

[51] Young BE (2020). Epidemiologic features and clinical course of patients infected with SARS-CoV-2 in Singapore. JAMA.

[52] Zhang Y (2020). Liver impairment in COVID-19 patients: A retrospective analysis of 115 cases from a single centre in Wuhan City, China. Liver Int. Off. J. Int. Assoc. Study Liver.

[53] Cai Q (2020). COVID-19 in a designated infectious diseases hospital outside Hubei Province. China..

[54] Xu, X. W. *et al.* Clinical findings in a group of patients infected with the 2019 novel coronavirus (SARS-Cov-2) outside of Wuhan, China: Retrospective case series. **368**, m606. 10.1136/bmj.m606 (2020). 10.1136/bmj.m606 PMC7224340 32075786

[55] Mo P (2020). Clinical characteristics of refractory COVID-19 pneumonia in Wuhan, China. Clin. Infect. Diseases Off. Publ. Infect. Diseases Soc. Am..

[56] Wang Z, Yang B, Li Q, Wen L, Zhang R (2020). Clinical features of 69 cases with coronavirus disease 2019 in Wuhan, China. Clin. Infect. Diseases Off. Publ. Infect. Diseases Soc. Am..

[57] Arentz M (2020). Characteristics and outcomes of 21 critically ill patients with COVID-19 in Washington State. JAMA.

[58] Zhu W (2020). Initial clinical features of suspected coronavirus disease 2019 in two emergency departments outside of Hubei. China. J. Med. Virol..

[59] Wan, S., Xiang, Y., Fang, W. & Zheng, Y. Clinical features and treatment of COVID-19 patients in northeast Chongqing. *J. Med. Virol* **92**(7), 797–806 10.1002/jmv.25783 (2020). 10.1002/jmv.25783 PMC7228368 32198776

[60] Shi H (2020). Radiological findings from 81 patients with COVID-19 pneumonia in Wuhan, China: A descriptive study. Lancet. Infect. Dis.

[61] Huang C (2020). Clinical features of patients infected with 2019 novel coronavirus in Wuhan, China. Lancet (London, England).

[62] Xu Z (2020). Pathological findings of COVID-19 associated with acute respiratory distress syndrome. Lancet Respir. Med..

[63] Yang W (2020). Clinical characteristics and imaging manifestations of the 2019 novel coronavirus disease (COVID-19): A multi-center study in Wenzhou city, Zhejiang, China. J. Infect..

[64] Wang D (2020). Clinical characteristics of 138 hospitalized patients with 2019 novel coronavirus-infected pneumonia in Wuhan, China. JAMA.

[65] Qian GQ (2020). Epidemiologic and clinical characteristics of 91 hospitalized patients with COVID-19 in Zhejiang, China: A retrospective, multi-centre case series. QJM Monthly J. Assoc. Phys..

[66] Qian ZP (2020). Analysis of baseline liver biochemical parameters in 324 cases with novel coronavirus pneumonia in Shanghai area. Chin. J. Hepatol..

[67] Yao N (2020). Clinical characteristics and influencing factors of patients with novel coronavirus pneumonia combined with liver injury in Shaanxi region. Zhonghua gan zang bing za zhi = Zhonghua ganzangbing zazhi = Chin. J. Hepatol..

[68] Huang Y (2020). Clinical characteristics of laboratory confirmed positive cases of SARS-CoV-2 infection in Wuhan, China: A retrospective single center analysis. Travel Med. Infect. Disease..

[69] Meszaros M (2020). Abnormal liver tests in patients hospitalized with Coronavirus disease 2019: Should we worry?. Liver Int. Off. J. Int. Assoc. Study Liver.

[70] Li S (2020). COVID-19 induced liver function abnormality associates with age. Aging (Albany NY).

[71] Hao SR (2020). Liver enzyme elevation in coronavirus disease 2019: A multicenter, retrospective, cross-sectional study. Am. J. Gastroenterol..

[72] Schattenberg JM (2020). Patterns of liver injury in COVID-19—A German case series. United Eur. Gastroenterol. J..

[73] Wang Q (2020). Pattern of liver injury in adult patients with COVID-19: A retrospective analysis of 105 patients. Mil. Med. Res..

[74] Fan Z (2020). Clinical features of COVID-19-related liver functional abnormality. Clin. Gastroenterol. Hepatol..

[75] Lei, F. *et al.* Longitudinal association between markers of liver injury and mortality in COVID-19 in China. *Hepatology* **72**(2), 389–398. 10.1002/hep.31301 (2020). 10.1002/hep.31301 PMC7267515 32359177

[76] Boeckmans J (2020). COVID-19 and drug-induced liver injury: A problem of plenty or a petty point?. Arch. Toxicol..

[77] Robinson MW, Harmon C, O'Farrelly C (2016). Liver immunology and its role in inflammation and homeostasis. Cell Mol. Immunol..

