# Supplementary material for: Effect of COVID-19 on liver abnormalities: a systematic review and meta‐analysis
Source: Sci Rep. 2021 May 19;11:10599. doi: 10.1038/s41598-021-89513-9 (PMC8134580; doi:10.1038/s41598-021-89513-9)

Effect of COVID-19 on Liver Abnormalities: A Systematic review and Meta-analysis

Khalid Bzeizi, FRCP, MD [1*], Maheeba Abdulla, MD [2], Nafeesa Mohammed, MD [3], Jehad Alqamish, MD [4], Negar Jamshidi, PhD [5], Dieter Broering, MD, PhD, FEBS, FACS[6]

[1]Department of Liver Transplantation, King Faisal Specialist Hospital & Research Center, P.O. Box 3354, Riyadh 11211, Saudi Arabia

[2,3]Internal Medicine Department, Salmaniya Medical Complex, Manama, Bahrain

[4]Ibn AlNafees Hospital, Manama, Bahrain

[5] School of Science, RMIT University, Melbourne, VIC 3000, Australia

[6]AlFaisal University, Organ Transplant Center & Department of Surgery, King Faisal Specialist Hospital & Research Centre (Gen. Org) MBC 96, P.O. Box 3354, Riyadh 11211, Saudi Arabia

Running title: Liver and Covid-19

***Corresponding author:**

Dr Khalid Bzeizi

Department of Liver Transplantation, King Faisal Specialist Hospital & Research Center,

P.O. Box 3354, Riyadh 11211, Saudi Arabia

T: +966115576162

Email: kbzeizi@kfshrc.edu.sa

Sensitivity analysis

(Supplementary Figure S1).

Alanine aminotransferase (ALT)

Study or Subgroup	Mean	SE	Weight	Mean IV, Random, 95% CI
Cai Q et al., 2020	43	1.75	5.2%	43.00 [39.57, 46.43]
Cai Q et al., 2020 (2)	22.23	0.68	5.6%	22.23 [20.90, 23.56]
Huang C et al. 2020	34.33	3.48	4.2%	34.33 [27.51, 41.15]
Lei F et al., 2020	26.03	0.23	5.7%	26.03 [25.58, 26.48]
Li X et al., 2020	28.83	4.64	3.4%	28.83 [19.74, 37.92]
Meszaros et al., 2020	10.4	10.2	1.4%	10.40 [-9.59, 30.39]
Mo P et al., 2020	25.67	1.32	5.4%	25.67 [23.08, 28.26]
Qian ZP et al., 2020	27.86	1.11	5.5%	27.86 [25.68, 30.04]
Shao-Rui Hao et al., 2020	9.85	1.8644	5.1%	9.85 [6.20, 13.50]
Shasha Li et al., 2020	11.75	1.1273	5.5%	11.75 [9.54, 13.96]
Shi H et al.2020	46.2	3.26	4.3%	46.20 [39.81, 52.59]
Wang D et al, 2020	26.67	1.53	5.3%	26.67 [23.67, 29.67]
Wang L et al., 2020	29.33	1.15	5.5%	29.33 [27.08, 31.58]
Wang Z et al., 2020	27.33	2.1	5.0%	27.33 [23.21, 31.45]
Wan S et al., 2020	24.02	1.31	5.4%	24.02 [21.45, 26.59]
Wu J et al., 2020	24.67	2.19	5.0%	24.67 [20.38, 28.96]
Xie H et al., 2020	83.67	7.96	1.9%	83.67 [68.07, 99.27]
Xu XW et al., 2020	23.33	1.93	5.1%	23.33 [19.55, 27.11]
Yang W et al., 2020	20	1.68	5.2%	20.00 [16.71, 23.29]
Zhang Y et al., 2020	25.71	1.97	5.1%	25.71 [21.85, 29.57]
Zhu W et al.,2020	25.2	1.89	5.1%	25.20 [21.50, 28.90]
**Total (95% CI)**			**100.0%**	**26.97 [24.30, 29.63]**

Heterogeneity: Tau² = 32.55; Chi² = 480.92, df = 20 (P < 0.00001); I² = 96%
Test for overall effect: Z = 19.80 (P < 0.00001)

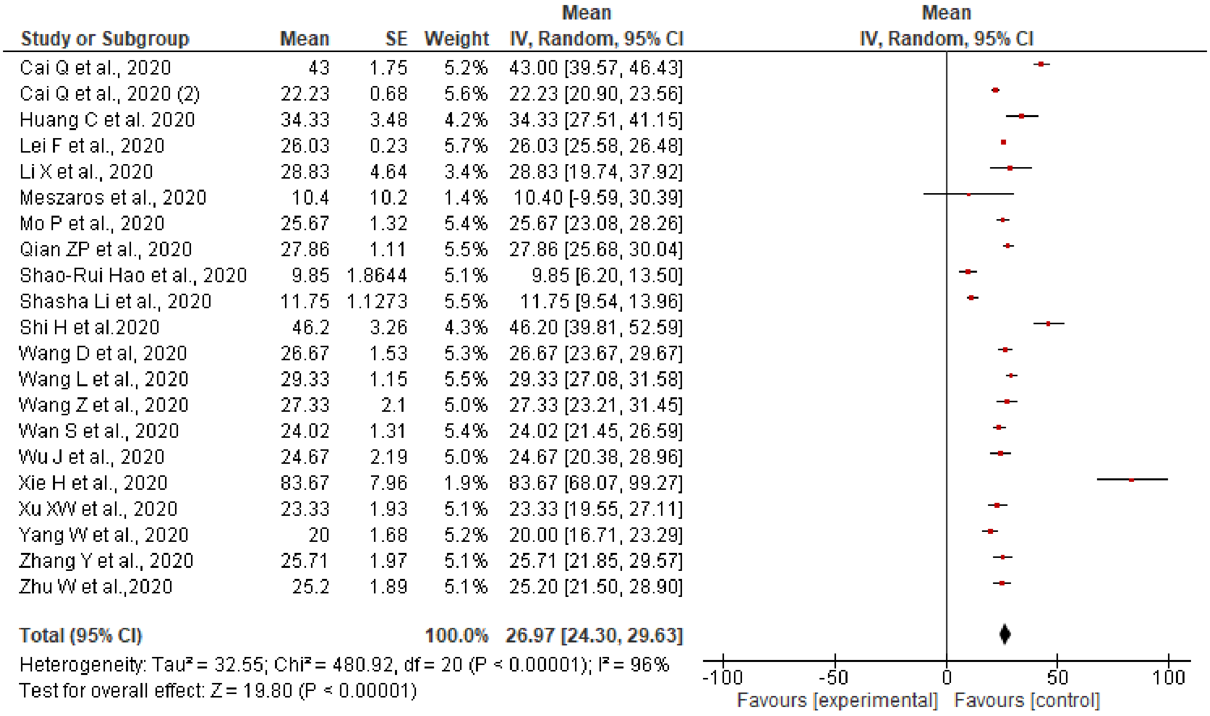

(Supplementary Figure S2).

Aspartate aminotransferase (AST)

Study or Subgroup	Mean	SE	Weight	Mean IV, Random, 95% CI
Cai Q et al., 2020	35.33	0.75	5.8%	35.33 [33.86, 36.80]
Cai Q et al., 2020 (2)	28.53	0.62	5.8%	28.53 [27.31, 29.75]
Huang C et al. 2020	36	2.64	4.7%	36.00 [30.83, 41.17]
Lei F et al., 2020	25.33	0.18	5.9%	25.33 [24.98, 25.68]
Li X et al., 2020	41.33	4.4	3.4%	41.33 [32.71, 49.95]
Meszaros et al., 2020	45	32	0.1%	45.00 [-17.72, 107.72]
Mo P et al., 2020	34.67	1.44	5.5%	34.67 [31.85, 37.49]
Qian ZP et al., 2020	29.33	1.17	5.6%	29.33 [27.04, 31.62]
Shasha Li et al., 2020	24	2.5981	4.7%	24.00 [18.91, 29.09]
Shi H et al.2020	40.8	1.98	5.1%	40.80 [36.92, 44.68]
Wang D et al, 2020	30.67	1.82	5.3%	30.67 [27.10, 34.24]
Wang L et al., 2020	35.33	1.72	5.3%	35.33 [31.96, 38.70]
Wang Z et al., 2020	33.67	0.93	5.7%	33.67 [31.85, 35.49]
Wan S et al., 2020	34.7	1.08	5.7%	34.70 [32.58, 36.82]
Wu J et al., 2020	29.33	1.69	5.3%	29.33 [26.02, 32.64]
Xie H et al., 2020	60	4.05	3.6%	60.00 [52.06, 67.94]
Xu XW et al., 2020	26	1.16	5.6%	26.00 [23.73, 28.27]
Yang W et al., 2020	23	1.29	5.6%	23.00 [20.47, 25.53]
Zhang Y et al., 2020	28.3	1.46	5.5%	28.30 [25.44, 31.16]
Zhu W et al.,2020	25.7	1.1	5.7%	25.70 [23.54, 27.86]
**Total (95% CI)**			**100.0%**	**32.01 [29.61, 34.42]**

Heterogeneity: Tau² = 25.41; Chi² = 530.41, df = 19 (P < 0.00001); I² = 96%
Test for overall effect: Z = 26.07 (P < 0.00001)

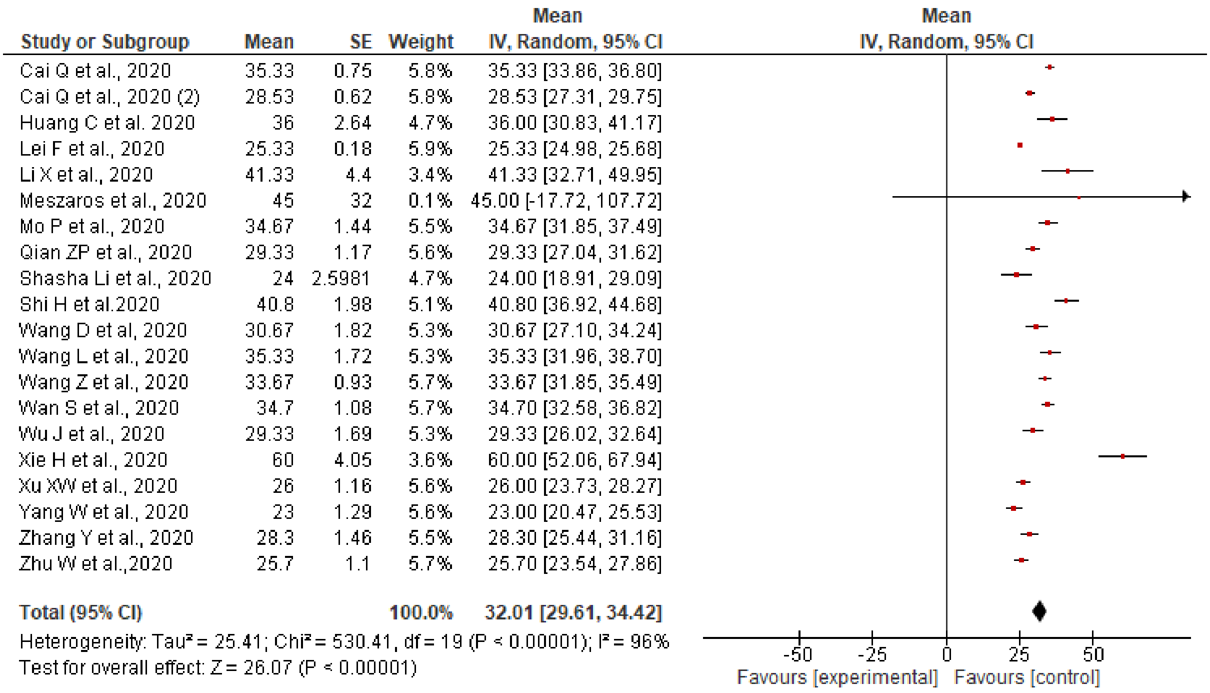

(Supplementary Figure S3).

Total Bilirubin (TB)

Study or Subgroup	Mean	SE	Weight	Mean IV, Random, 95% CI
Arentz M et al., 2020	0.6	28.03	0.0%	0.60 [-54.34, 55.54]
Cai Q et al., 2020	19.33	0.54	7.4%	19.33 [18.27, 20.39]
Cai Q et al., 2020 (2)	10.9	0.85	6.6%	10.90 [9.23, 12.57]
Huang C et al. 2020	11.7	0.53	7.5%	11.70 [10.66, 12.74]
Lei F et al., 2020	10.8	0.06	8.1%	10.80 [10.68, 10.92]
Meszaros et al., 2020	10.4	10.2	0.3%	10.40 [-9.59, 30.39]
Qian ZP et al., 2020	9.46	0.25	7.9%	9.46 [8.97, 9.95]
Shao-Rui Hao et al., 2020	9.85	1.8644	4.0%	9.85 [6.20, 13.50]
Shasha Li et al., 2020	11.75	1.1273	5.8%	11.75 [9.54, 13.96]
Shi H et al.2020	11.9	0.41	7.7%	11.90 [11.10, 12.70]
Wang D et al, 2020	10.77	0.36	7.8%	10.77 [10.06, 11.48]
Wan S et al., 2020	9.4	0.5	7.5%	9.40 [8.42, 10.38]
Wu J et al., 2020	8	0.56	7.4%	8.00 [6.90, 9.10]
Xie H et al., 2020	13.32	0.75	6.9%	13.32 [11.85, 14.79]
Yang F et al., 2020	160.08	34.14	0.0%	160.08 [93.17, 226.99]
Yang W et al., 2020	9.9	0.49	7.5%	9.90 [8.94, 10.86]
Zhang Y et al., 2020	11.31	0.48	7.6%	11.31 [10.37, 12.25]
**Total (95% CI)**			**100.0%**	**11.36 [10.35, 12.38]**

Heterogeneity: Tau² = 3.33; Chi² = 357.09, df = 16 (P < 0.00001); I² = 96%
Test for overall effect: Z = 21.90 (P < 0.00001)

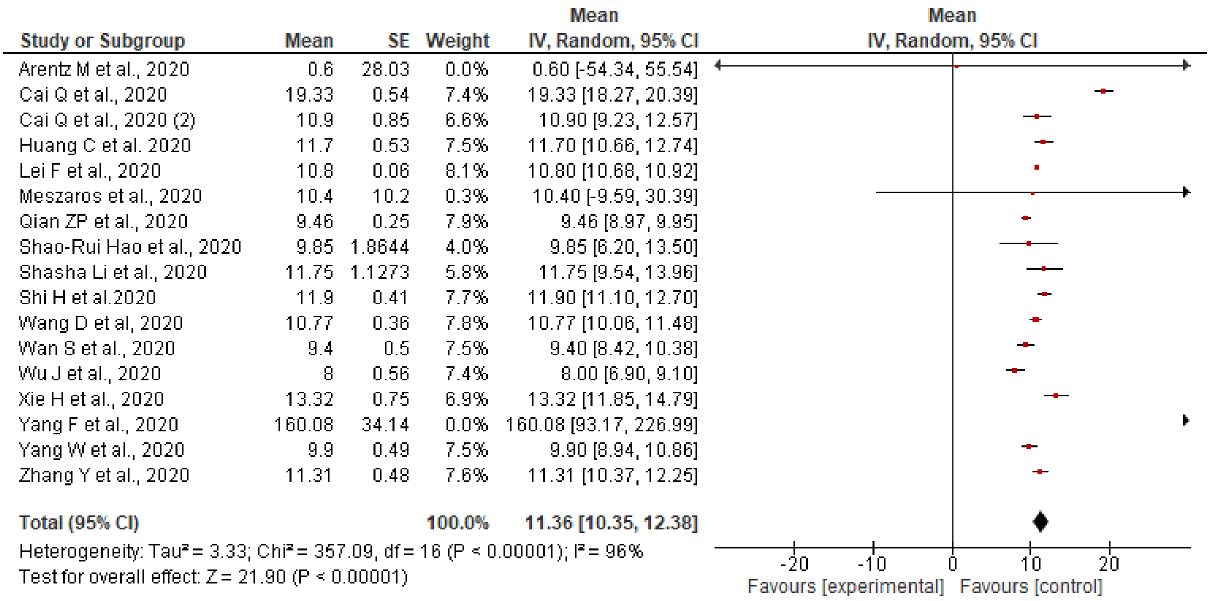

(Supplementary Figure S4).

Study or Subgroup	Mean	SE	Weight	Mean IV, Random, 95% CI
Cai Q et al., 2020	19.33	0.54	7.9%	19.33 [18.27, 20.39]
Cai Q et al., 2020 (2)	11.67	0.33	8.4%	11.67 [11.02, 12.32]
Huang C et al. 2020	11.7	0.53	7.9%	11.70 [10.66, 12.74]
Lei F et al., 2020	10.8	0.06	8.7%	10.80 [10.68, 10.92]
Meszaros et al., 2020	10.4	10.2	0.2%	10.40 [-9.59, 30.39]
Qian ZP et al., 2020	9.46	0.25	8.5%	9.46 [8.97, 9.95]
Shao-Rui Hao et al., 2020	9.85	1.8644	3.9%	9.85 [6.20, 13.50]
Shasha Li et al., 2020	11.75	1.1273	6.0%	11.75 [9.54, 13.96]
Shi H et al.2020	11.9	0.41	8.2%	11.90 [11.10, 12.70]
Wang D et al, 2020	10.77	0.36	8.3%	10.77 [10.06, 11.48]
Wan S et al., 2020	9.4	0.5	8.0%	9.40 [8.42, 10.38]
Wu J et al., 2020	8	0.56	7.8%	8.00 [6.90, 9.10]
Yang W et al., 2020	9.9	0.49	8.0%	9.90 [8.94, 10.86]
Zhang Y et al., 2020	11.31	0.48	8.0%	11.31 [10.37, 12.25]
**Total (95% CI)**			**100.0%**	**11.25 [10.27, 12.22]**

Heterogeneity: Tau² = 2.86; Chi² = 333.33, df = 13 (P < 0.00001); I² = 96%
Test for overall effect: Z = 22.57 (P < 0.00001)

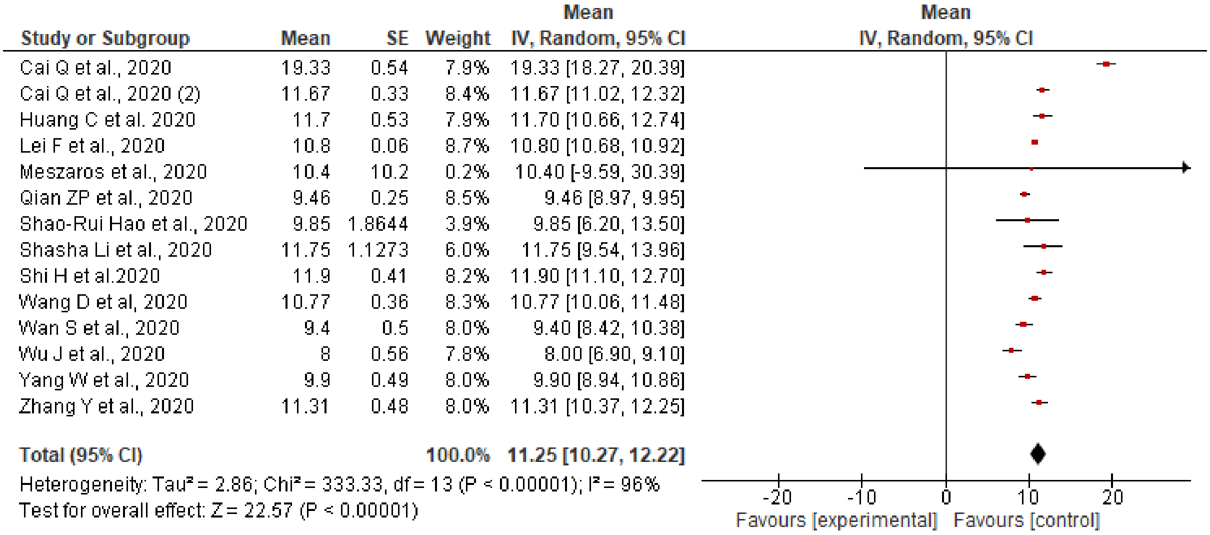

Supplement: Supplementary file 2 — Supplementary Information 2. [file 41598_2021_89513_MOESM2_ESM.pdf]
